# Effects of Antioxidants in Human Cancers: Differential Effects on Non-Coding Intronic RNA Expression

**DOI:** 10.3390/antiox5010001

**Published:** 2016-01-04

**Authors:** Shreya Menon, Chunxia Lu, Rajasree Menon, Jessica Schwartz, Yuanfang Guan

**Affiliations:** 1 Skyline High School, Ann Arbor, MI-48103, USA; mnnshreya@gmail.com; 2 Department of Pediatrics, University of Michigan, Ann Arbor, MI-48109, USA; chunxial@med.umich.edu; 3 Department of Computational Medicine and Bioinformatics, University of Michigan, Ann Arbor, MI-48109, USA; rajmenon@umich.edu; 4 Department of Molecular and Integrative Physiology, University of Michigan, Ann Arbor, MI-48109, USA; Jeschwar@med.umich.edu

**Keywords:** antioxidants, intronic RNA, lung cancer, prostate cancer

## Abstract

The notion that dietary antioxidants can help fight cancer is popular. However, the mechanism(s) behind the effect of antioxidants in cancer is still unclear. Previous studies indicate that supplements can influence gene expression; however, all of these studies were focused on the coding/exonic gene expression. Studies are now emerging to highlight critical functional roles for RNAs expressed from the non-coding regions. This project was designed to study the effect of antioxidant supplements on non-coding intronic RNA expression in human cancers. Vitamin E, *N*-Acetyl cysteine (NAC) and Sulforaphane are commonly used supplements to prevent diseases including cancers. We studied the effect of these antioxidant supplements on the non-coding intronic RNA expression using publicly available datasets from a mouse model for lung cancer and prostate cancer cell lines. Although high throughput polyA-enriched RNA-Seq data characterize spliced coding mRNA regions, recent studies reveal the expression of reads from the non-coding intronic regions. Our analyses indicate that cancer cells have higher expression of introns compared to that of normal cells and that treatment with antioxidant supplements reduces the increased expression of introns of several genes. However, we did find high expression of introns of multiple genes including many oncogenes in the supplement treated groups compared to that of the control; this effect was distinct depending on the cell type and the supplement studied. Using RT-PCRs, we validated the expression of introns of two oncogenes, DLK1 and LRG1, known to be key players in lung cancer progression, and demonstrate changed intronic expression with supplement treatment in cancer cells. With regard to the antioxidant system, supplements did not change the intronic RNAs for endogenous antioxidant enzymes except for a significant decrease in the expression of superoxide dismutase (SOD) intronic RNA. Concurrently, we also found that a prolonged (48 h) exposure to Vitamin C, Vitamin E and Green tea extract reduced the enzymatic activity of SOD in lung cancer cells. The results from this study reveal that the antioxidant supplements have a significant effect on the intronic RNA expression of many genes including cancer genes that are not directly linked to the body’s antioxidant system. It is important to study this novel effect of antioxidant supplements in detail as it may have a significant role in disease progression.

## 1. Introduction

Reactive oxygen species (ROS) are generated in the body as byproducts of several cellular metabolic reactions; they consist of radical and non-radical oxygen species formed by the partial reduction of oxygen [1] Low levels of ROS are necessary for cellular processes such as intracellular signaling, cell progression and cell defense. Conversely, high levels of the ROS or the inability of the antioxidant system to regulate ROS levels efficiently results in oxidative stress. Oxidative stress results in direct or indirect ROS-mediated damage of nucleic acids, proteins, and lipids [1]. Two examples of reactions that generate ROS (hydrogen peroxide) are the conversion of lipids and fat into carbohydrates, another component of food, and the conversion of amino acids into glucose, the basic “fuel” for the body. To prevent a buildup of ROS, the body produces defenders that extinguish ROS as soon as they are produced; these defenders or molecules are collectively known as “antioxidants”. The antioxidant system consists of the enzymatic defenses, such as superoxide dismutase (SOD), glutathione peroxidase (GPX) and catalase (CAT), as well as the non-enzymatic defenses ascorbic acid (Vitamin C), a-tocopherol (Vitamin E), and B-carotene. The balance and efficiency of this system is essential for the health of the organism [2].

Consuming dietary antioxidant supplements to fight diseases, especially cancer, has become popular among the general public. However, the exact mechanism(s) for the effect of antioxidants in cancer is unclear. In addition clinical trials have reported inconsistent outcomes. Hennekens *et al.* [3] reported that twelve years of beta carotene supplementation in healthy men did not produce benefit in terms of incidence of malignant neoplasms and cardiovascular diseases. Conversely, Herceberg *et al.* reported that low-dose antioxidant supplementation for 7.5 years lowered total cancer incidence and all-cause mortality in men but not in women [4].

Several studies including state of the art transcriptomic expression analysis using RNA-Sequencing (RNA-Seq), have reported on the effects of antioxidants on gene expression [5,6,7,8]. According to Matsumoto *et al.*, antioxidant supplementation up-regulated the cardiac endothelial cell gene expression of the ras homolog gene family member A, which has a role in cardiovascular disease progression [9]. In another study, dietary glutamine supplementation decreased oxidative stress-related gene expression, increased the antioxidant potential and attenuated renal oxidative damage in rats with streptozotocin-induced diabetes [10].

Until the late 2000s, the focus of gene expression studies was mainly on coding mRNA expression. It was previously believed that RNA transcribed from the intronic regions was destined to be degraded and hence did not have a biological function. However, in recent years, studies have highlighted critical functional roles for these non-coding regions [11]. Martianov *et al.* experimentally validated the repression of the human dihydrofolate reductase gene by a non-coding interfering transcript [12]. Subsequently, several studies have reported on the role of non-coding intronic RNAs in diseases. Brito *et al.* reported that in renal cell carcinoma, the expression of non-coding intronic RNA discriminates tumor from adjacent non-malignant cells [13]. Similarly, Reis *et al.* showed that the expression of six antisense intronic non-coding RNA transcripts correlated with the degree of tumor differentiation in prostate cancer [14]. However, to the best of our knowledge, previous studies have not investigated the effect of antioxidant supplements on non-coding RNA expression in cancers. This project examined the effect of antioxidant supplements on non-coding intronic RNA expression in cancers.

The hypothesis for this study was that antioxidant supplements would influence the expression of intronic RNAs in genes linked to ROS system. We utilized two publicly available RNA-Seq datasets for studying the effect of antioxidant supplements on intronic RNA expression. The antioxidants studied in these datasets were Vitamin E, *n*-Acetyl cysteine and Sulforaphane. The rationale for choosing these data sets was that the datasets were of good quality and that the antioxidant supplements studied are commonly used, while research on the effects of these supplements has yielded contradictory results [15,16,17,18,19,20].

For both RNA-Seq datasets, prior to sequencing, the RNA samples were enriched for polyadenylated RNA fractions containing processed spliced RNA transcripts. Polyadenylated RNA-Seq data should generate reads that map to exons. However, it has been shown that RNA-Seq data also capture intronic sequences [21]. The interpretation for the expression of intronic RNA has remained controversial. Some have suggested that the intronic reads originate from DNA contamination or nascent RNAs whereas others report that they are unknown exons or intronic enhancers [21,22]. Gaidatzis *et al.* show that most intronic reads arise from nuclear RNA and changes in intronic read counts accurately predict changes in transcriptional activity [23]. In the present study we demonstrate that antioxidant supplements cause significant differential intronic RNA expression in several genes linked to cancer-related processes. Furthermore, we experimentally validate the expression of introns of two oncogenes, DLK1 and LRG1, which are major players in lung cancer progression [24,25].

## 2. Materials and Methods

**Figure 1:**
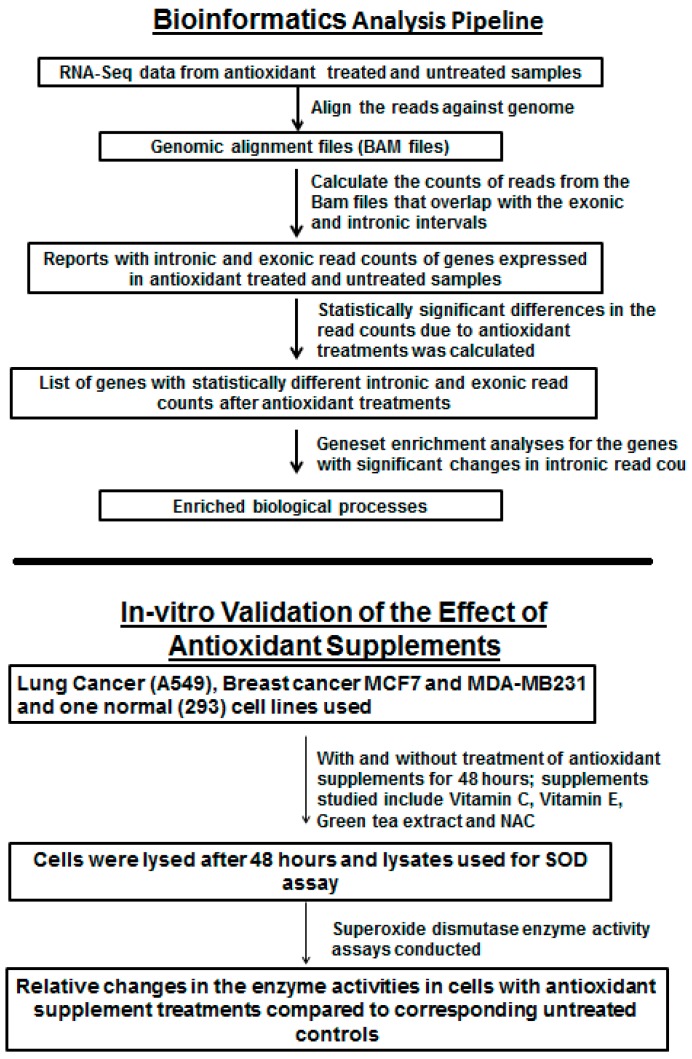
Experimental strategy.

The analysis approach used in this study is shown in Figure 1. Based on our hypothesis, we focused our analyses on non-coding intronic RNA expression in normal and cancer samples supplemented with antioxidants. We used the publicly available datasets shown in Table 1.

### 2.1. RNA-Seq Data Analysis

RNA-Seq is a relatively recent approach to transcriptome profiling that uses deep-sequencing technologies which provide a more precise measurement of levels of transcripts and their isoforms than traditional microarrays [26]. The RNA-Seq data (Table 1) were downloaded from Array Express and NCBI SRA. The datasets downloaded include.

1. Murine lung cancer dataset (Accession: E-GEOD-52594): RNA-Seq data were downloaded from ArrayExpress. The objective of the original study was to study the impact of Vitamin E and NAC supplementation in murine models of KRAS-induced lung cancer [27]. Antioxidants were administered 1 week after the induction of lung cancer, and the mice were euthanized 8 to 10 weeks later. There were 3 experimental groups (untreated, NAC-treated and Vitamin E-treated. Each group consisted of 5 animals, and from each animal two tumor samples were harvested for analysis. A total of 30 samples were profiled by RNA-Seq analyses.
2. Human normal and prostate cancer cells dataset (SRP027258): RNA-Seq data were downloaded from NCBI SRA. Normal prostate epithelial cells and androgen-dependent and androgen-independent prostate cancer cells were treated with 15 µM sulforaphane (SFN), a phytochemical derived from cruciferous vegetables, and the transcriptome was determined at 6 and 24 h time points [28].

The mouse and human genome (Ensembl version) files were downloaded from iGenomes [29]. The RNA-Seq data sequence mapping was done using Tophat2 [30]. Tophat2 which aligns RNA-Seq reads to genomes using the short read aligner Bowtie [31]. Default parameters were used in Tophat2 for mapping. The outputs from these analyses are BAM files.

**Table 1:** RNASeq Datasets Downloaded from NCBI-SRA/ArrayExpress for Analyses.

Antioxidant Supplements Studied	Organism	NCBI/Arrayexpress Accession	Brief Description of The Original Study
Vitamin E, *N*-acetyl cysteine (NAC)	Mus musculus (mouse)	E-GEOD-52594	The original study [27] found that both antioxidants increase tumor cell proliferation by reducing ROS, DNA damage, and p53 expression.
Sulforaphane (SFN)	Human prostate normal and cancer cell lines	SRP027258	SFN influenced the expression of genes in functional groups and pathways that are critical in cancer including cell cycle, apoptosis and angiogenesis, but the specific effects of SFN differed depending on the state of cancer progression [28].

### 2.2. Intronic Count Analysis

The intronic counts were calculated from BAM files using the multicov function in samtools [32]. The intronic co-ordinate files for mapping the reads were downloaded from UCSC Table Browser [33]. The differentially expressed genes based on intronic read counts were done using EdgeR package in R [34]. This method uses an over-dispersed Poisson model to account for biological and technical variability. Multiple hypotheses corrections (Bonferroni correction) to the *p* values of the differentially expressed genes were done using p.adjust function in stats, R package [35]. Geneset enrichment tool [36] was used for functional enrichment analyses of the differentially expressed genes.

### 2.3. In Vitro Analysis on the Effect of Antioxidant Supplements on Superoxide Dismutase Activity on Established Human Cell Lines

The results from the bioinformatic analyses indicated that the antioxidants decreased the activity of one of the major group of antioxidant enzymes, superoxide dismutases. We conducted *in vitro* analyses to experimentally validate the effect of antioxidants on superoxide dismutase activity. The cell lines used in this study were obtained from ATCC (www.atcc.org). We used A549 (lung cancer), MCF-7 (breast cancer), MDA-MB231 (breast cancer) and 293 (normal embryonic kidney) cell lines for studying the effect of antioxidant supplements including green tea extract, Vitamin C, Vitamin E and n-Acetyl cysteine. The antioxidants were obtained from and manufactured by the Vitamin Shoppe. The non-toxic dosages for the antioxidants: 2 mM Vitamin C, 25 µM Vitamin E2, 50 µg/mL Green tea extract and 0.2 mg/mL *N*-acetyl cysteine were selected from previously published studies [37,38,39,40]. The cell lines were exposed to media supplemented with each antioxidant individually for 48 h. For each cell line, there was one control and four experimental (treated) groups. After 48 h, cells were harvested, lysed with the cell lysis buffer, centrifuged and the supernatant was stored at −80 degrees pending the enzyme assays. Prior to the enzyme assay, a BioRad protein assay was performed to determine the protein concentration in each lysate [41]. Superoxide dismutase activity was assayed using the kit obtained from Cayman Chemicals, Ann Arbor, MI, USA following the manufacturer’s protocol [42]. The calculated enzyme activity per sample was normalized using the protein concentration of the respective sample.

### 2.4. RNA-Isolation and RT-PCR

To validate intronic RNA expression, we examined DLK1and LRG1 using RT-PCR. For the *in vitro* study of antioxidant enzyme activity, we had treated NAC and Vitamin E on A549 lung cancer and MCF7 breast cancer cells. We used the cell lysates from antioxidant treated and untreated A549 lung cancer and MCF7 breast cancer cells for RT-PCR validations.

The primers for DLK1 and LRG1 were designed using the software Primer3 [43]. QIAGEN’s RNeasy mini kit was used to extract total RNA from cell culture lysates. Real-time quantitative PCR was performed using Invitrogen’s OneStep RT_PCR kit. PCRs were done in duplicate for each sample type. Forward and reverse primers for DLK1 (182 bp): AGTCTGGGGTAGGGGAAAGA and GACCGTCACTTTTGCAACCT; LRG1 (215 bp): GCACCCAATTGGTCAAGAGT and CTAGCCCCCATGAGCTGTTA. GAPDH (Applied Biosystems, Warrington, United Kingdom) was used as reference gene; the fragment size of the amplified GAPDH was 173 bp.

## 3. Results

The summary of genes with significantly differentially expressed (FDR < 0.01 and fold change >2) intronic regions in the antioxidant supplement treated tumor groups compared to the untreated tumor group is given in Table 2. The complete list of genes with differentially expressed intronic RNA in the two dataset analyses is given in Supplementary Data 1 and 2. The numbers of genes where all the introns of the gene were significantly differentially expressed in NAC and Vitamin E treated groups were 35 and 149, respectively. In other instances the differential intronic RNA expression was detected only in certain introns of the gene (Supplementary Figure S1).

### 3.1. Murine Lung Cancer Dataset

**Differentially expressed intronic RNA:** The summary of the results from the analyses of murine lung cancer dataset is given in Table 2a. The antioxidant supplement treatments tend to reduce the intronic RNA expression as evident by the higher number of genes with significantly reduced expression (Table 2a). The numbers of genes with differentially expressed intronic RNA were much greater in the Vitamin E treated group compared to NAC-treated group.

**Table 2:** Summary of the total number of genes with significant differential expression of introns (FDR < 0.01 and fold change >2 (up or down)).

a. Summary of Results from Murine Model for KRAS-Induced Lung Cancer Data Analysis										
Total Number of Genes with Significant Differences in the Intronic Read Counts from Antioxidant Supplement (NAC, Vitamin E) Treated Tumor Tissues Compared to Control Tumor Tissue										
		NAC					Vitamin E			
		Down		Up			Down		Up	
Number of genes		459		86			1143		315	
**b. Summary of Results from Human Prostate Cancer Cells Data Analysis**										
Total Number of Genes with Significant Differences in the Intronic Read Counts in Sulforaphane Treated Cells Compared to Untreated Cells										
	Normal Prostate Epithelial Cells				LNCAP (Prostate Cancer Cells, Hormone, Dependent)			PC3 (Prostate Cancer Cells, Hormone Independent)		
	Down		Up		Down	Up		Down		Up
6 h	1680		1971		428	575		569		707
24 h	1522		1437		1313	476		496		457

**Antioxidant enzymes:** Since we wanted to know if the supplements would have an effect on the ROS-system, we also studied the changes in expression in the major antioxidant enzymes including, catalase (CAT), glutathione peroxidases (GPXs) and superoxide dismutases (SODs). The exonic expression of these antioxidant enzymes was generally reduced after supplement treatments and a significant decrease was observed after Vitamin E treatment. Furthermore, we found significant decrease in intronic RNA expression of SOD2 after Vitamin E supplementation.

### 3.2. Functional Annotation of Genes with Differentially Expressed Intronic RNA

**NAC supplement:** According to Gene set enrichment analyses, out of a total 86 genes with significantly increased intronic RNA expression in the NAC supplemented murine model group, 16 overlapped with the gene-expression signature of KRAS2 mutation in human lung cancer [44] (Table 3).

**Table 3:** List of genes with increased intronic RNA expression after Vitamin E supplement treatment that overlapped with the genes in the oncogenic KRAS2 expression signature. The gene symbols with asterisk also had increased expression of introns after NAC supplement treatment.

Gene Symbols				
ALDOC	F7 *	IL18	MRC1 *	SOCS3
AREG	FCGR2B	ITGAX *	MSR1	STARD10
AXL	GAPDH	ITGB2	PTGS1	TNFSF9
CAMSAP1	GJA1	ITIH4 *	ROS1 *	TYROBP
CD68	HDC	KRAS	RPL3	
CRLF1	HK1 *	LCP1	SERPINE1	
CTSK	HK2 *	LRG1 *	SH3RF1	
ELL2	HSPA1B	LRP2 *	SIRPA	
EPHA7	HSPA8	ME1	SLAIN1	
F10	HSPH1	MMP12	SLC38A2	

The top five pathways after the enrichment analyses of the genes with differential expression of introns after NAC supplement treatment are shown in Table 4.

**Table 4:** Top 5 enriched pathways for the genes with differentially expressed introns after NAC supplement treatment.

For Genes with Increased Expression of Introns
Fructose and mannose metabolism
Ensemble of genes encoding ECM-associated proteins including ECM-affiliated proteins, ECM regulators and secreted factors
Ensemble of genes encoding extracellular matrix and extracellular matrix-associated proteins
Genes involved in Transmembrane transport of small molecules
Type II diabetes mellitus
**For Genes with Decreased Expression of Introns**
Drug metabolism—cytochrome P450
Genes involved in Biological oxidations
Genes involved in Muscle contraction
Metabolism of xenobiotics by cytochrome P450
Glutathione metabolism

**Figure 2:**
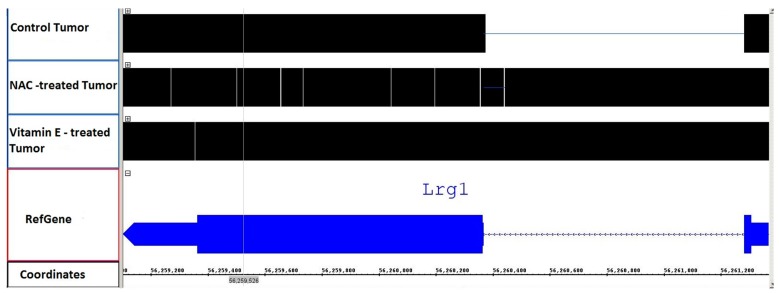
Illustration of genomic alignment of reads mapping to LRG1. For LRG1 gene, the exonic regions of the gene are shown as blue blocks and the intronic region as blue line (two exons and one intron). The regions with reads mapping to the gene are shown as black blocks. Both NAC and Vitamin E treated groups had reads mapping to the single intronic region compared to that of untreated control tumor tissue.

**Vitamin E supplement:** Table 3 shows the list of 44 genes with increased intronic RNA expression after Vitamin E supplement treatment that overlapped with the gene-expression signature of human lung cancer with KRAS2 mutation [44]. Immune system, extracellular matrix and transmembrane transport were the top gene sets from the enrichment analyses of genes with increased expression of introns after Vitamin E treatment. Similar to NAC treatment, drug metabolism via cytochrome p450 was among the top-ranking pathway for the genes with decreased expression of introns after Vitamin E supplement treatment (Table 5). Figure 2 shows genomic alignment of reads mapping to Leucine-Rich Alpha-2-Glycoprotein 1 (LRG1). Both NAC and Vitamin E supplement treatments increased the intronic RNA expression as shown in Figure 2.

**Table 5:** Top 5 enriched pathways for the genes with differentially expressed introns after Vitamin E supplement treatment.

Enriched Canonical Pathways
**For genes with Increased Expression of Introns**
Ensemble of genes encoding extracellular matrix and extracellular matrix-associated proteins
Genes involved in Adaptive Immune System
Beta2 integrin cell surface interactions
Ensemble of genes encoding ECM-associated proteins including ECM-affiliated proteins, ECM regulators and secreted factors
Genes involved in Transmembrane transport of small molecules
**For Genes with Decreased Expression of Introns**
Drug metabolism—cytochrome P450
Genes involved in Biological oxidations
Metabolism of xenobiotics by cytochrome P450
Ensemble of genes encoding extracellular matrix and extracellular matrix-associated proteins
Genes involved in Phase 1—Functionalization of compounds

**Signaling pathways:** Gene Set enrichment of genes with increased expression of introns after Vitamin E supplementation showed genes enriched in signaling pathways including beta 2 integrin, CXCR4, MAPK and TOLL-like receptor pathways. No signaling pathways were enriched in Gene Set enrichment analysis of the 86 genes, which showed increased expression of introns after NAC supplementation.

### 3.3. Human Normal and Prostate Cancer Cells Dataset

**Figure 3:**
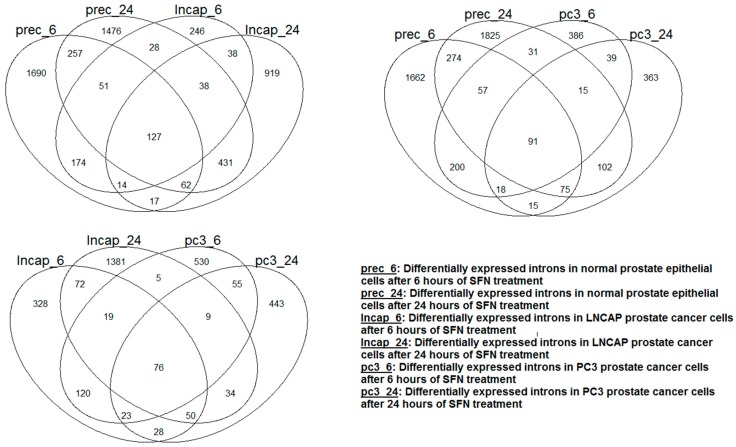
Venn diagram showing the overlap of the introns that were differentially expressed after SFN treatment in each cell type. The overlap was small between the cell types regardless of the cell type and treatment period.

**Differentially expressed intronic RNAs:** The summary of the results from the differential expression analyses is shown in Table 2b. The effect of SFN supplement treatment on the expression of introns was distinct depending on cell type and treatment time. This is evident in Figure 3, which shows the Venn diagram of the differentially expressed introns; each cell type had a large number of differentially expressed introns that were unique to them. More genes with differentially expressed introns after SFN treatment were observed in normal epithelial cells compared to that of the two prostate cancer cell lines. In contrast to PC3 cancer cells, exposure of LNCAP cancer cells to SFN tended to decrease the intronic RNAs of several genes.

The exonic expression of the three main antioxidant enzyme groups (CAT, GPXs and SODs) did not show any significant change after SFN treatment. However, significant decreases in SOD2 expression of introns were observed in LNCAP and PC3 prostate cancer cells.

**Differential Expression analyses:** The results from the differential expression analyses of the three prostate cell types after SFN treatment are shown in Table 2b. Sulforaphane (SFN) treatments for 6 and 24 h resulted in significant intronic differential expression (FDR < 0.01 with two-fold expression change) of many genes in normal prostate epithelial cells; increased in 1971 and 1437 genes and decreased in 1680 and 1522 genes, respectively (Table 2b). In LNCAP cells, the number of genes with increased expression of introns tripled after 24 h of SFN treatment compared to that of 6 h (Table 2a). In PC3 cells, a smaller number of genes had significantly differentially expressed intronic RNA compared to that of normal prostate epithelial cells (Table 2b).

The top canonical pathways of the genes with differentially expressed introns after SFN treatment in the three prostate cell types are shown in Table 6.

**Table 6:** Top 5 enriched canonical pathways for the genes with differentially expressed introns after SFN treatment.

Enriched Canonical Pathways
**Normal Prostate Epithelial Cells with 6 h of SFN Treatment**
Ensemble of genes encoding extracellular matrix and extracellular matrix-associated proteins
Genes involved in Developmental Biology
Genes involved in Transmission across Chemical Synapses
Genes involved in Immune System
Genes involved in Axon guidance
**Normal Prostate Epithelial Cells with 24 h of SFN Treatment**
Genes involved in Transmembrane transport of small molecules
Arrhythmogenic right ventricular cardiomyopathy (ARVC)
Focal adhesion
Genes involved in Immune System
Genes involved in Developmental Biology
**LNCAP Prostate Cancer Cells with 6 h of SFN Treatment**
Caspase cascade in apoptosis
Endocytosis
Genes involved in Developmental Biology
Peroxisome
Genes involved in Signaling by Rho GTPases
**LNCAP Prostate Cancer cells with 24 h of SFN Treatment**
Genes involved in Collagen formation
Genes involved in Developmental Biology
Genes involved in Neuronal System
Genes involved in Metabolism of lipids and lipoproteins
Genes involved in Extracellular matrix organization
**PC3 Prostate Cancer Cells with 6 h of SFN Treatment**
Genes involved in Collagen formation
Genes involved in Extracellular matrix organization
Regulation of RhoA activity
Genes involved in Metabolism of lipids and lipoproteins
Ensemble of genes encoding extracellular matrix and extracellular matrix-associated proteins
**PC3 Prostate Cancer Cells with 24 h of SFN Treatment**
Genes involved in Axon guidance
Genes involved in Immune System
Genes involved in NRAGE signals death through JNK
Genes involved in Signaling by Rho GTPases
Genes involved in Signaling by NGF

### 3.4. Validation of Intronic RNA in DLK1 and LRG1

**Figure 4:**
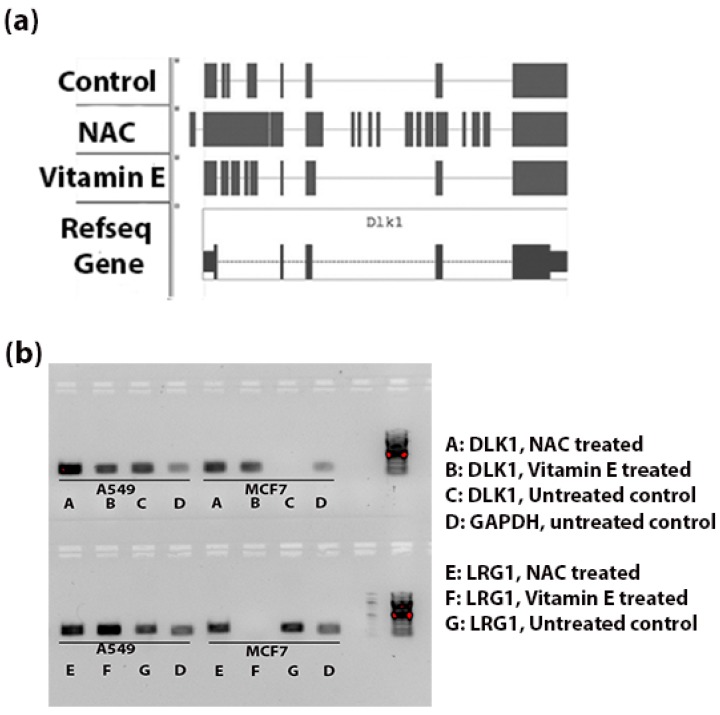
Intronic RNA expression of DLK1 and LRG1 (**a**) Schematic diagram to show the alignment of reads in the BAM files (Tophat output from the genomic alignment of RNA-seq data). Comparatively more reads are aligned to the intronic region (thin blue line) in the NAC-treated group than the control tumor and Vitamin E-treated groups. (**b**) RT-PCR validation of intronic RNA of DLK1 and LRG1 genes. Expression of introns of these two genes was observed in A549 and MCF7 cells. In A549 cells, NAC treatment seems to increase the expression of intronic RNA of DLK1; similarly the expression of introns of LRG1 was strong for the vitamin E treated A549 cells. No PCR amplification was observed with DLK1 intronic primers in the MCF7 control group and with LRG1 intronic primers in the MCF7 vitamin E treated group. The amplicon sizes of DLK1, LRG1 and GAPDH were 183 bp, 215 bp and 173 bp, respectively.

Figure 4a shows the genomic alignments of the RNA-seq reads against the exonic and intronic regions of DLK1 gene. NAC treatment resulted in significant increase (FDR < 0.003) in expression of introns of DLK1. Whereas Vitamin E treatment increased the expression of introns compared to control tumor tissue, this difference was not statistically significant (*p* < 0.3). Figure 4b indicates the intronic RNA expression in A549 and MCF7 using RT-PCR; the DLK1 expression of introns was comparatively stronger in the NAC treated A549 cells and LRG1 expression of introns was more in the vitamin E treated A549 cells.

**Figure 5:**
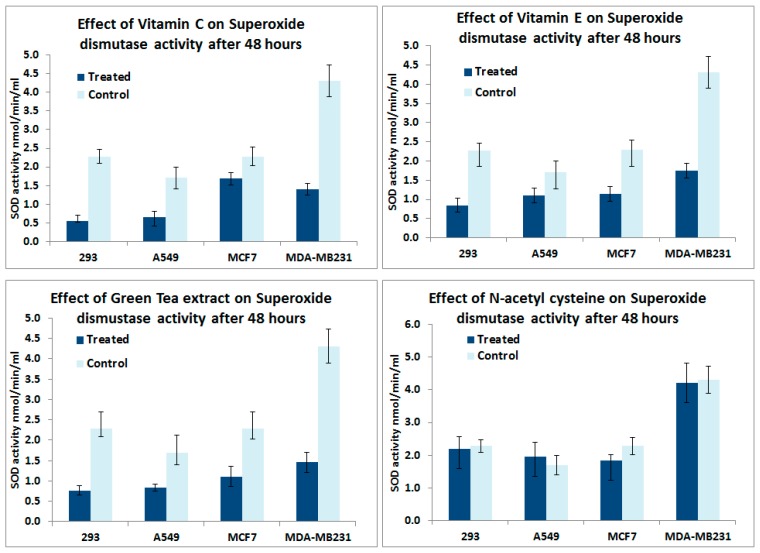
*In vitro* analyses on the effect of antioxidant supplements for 48 h on Superoxide dismutase activity in 293, MCF-7, MDA-MB231 and A549 lung cancer cells.

***In vitro* analyses of antioxidant supplements on superoxide enzyme activity:** Figure 5 shows the effect of Vitamin C, Vitamin E, Green tea extract and NAC on superoxide dismutase activity in normal 293, MCF-7, MDA-MB231 and A549 lung cancer cells. Vitamin C, Vitamin E and Green tea extract treatments for 48 h reduced the SOD enzyme activity in all cell types. However, we did not observe a significant change in the enzyme activity in all cell types after NAC treatments.

## 4. Discussion

In recent years, owing in part to increased media attention, the popularity and use of antioxidant supplements as health adjuvants has been rising. However, studies showing both beneficial and harmful effects of antioxidant supplements have been reported [45,46,47]. The exact mechanisms by which antioxidants act on the human body are not yet clearly known. In the present study using bioinformatics analysis we found significant differential effects of antioxidant supplements on intronic RNA expression in human cancer cells. Two other interesting observations include: (a) for many genes the differential expression was intron-specific; *i.e.*, not all introns were differentially expressed and (b) distinct signaling pathways were enriched for the genes with increased expression of introns after Vitamin E and SFN treatments.

Several recent studies clearly demonstrate the identification of functional intronic RNAs from RNA-Seq data [13,14,23]. This is in contrast to earlier thoughts that the presence of intronic RNA is either due to contamination of genomic DNA in the RNA library prep or from unspliced nascent RNA. In our analyses, we found many genes with significantly increased intronic RNA expression without increase in corresponding exonic RNA after the antioxidant supplementation. It is difficult to explain this phenomenon due to genomic DNA leakage or by the unspliced nascent RNA. The expression of intronic regions could be due to antisense mRNA expression or intronic retention. However, we were not able to discern antisense mRNA expression, as both of the RNA-Seq datasets we used in this study were not strand-specific. In malignancy, normal cellular processes including cell cycle, splicing and metabolism are altered [48]. Changes in splicing could be one of the mechanisms responsible for the expression of introns.

Intronic RNA has been shown to play roles in development [49], transcription regulation and alternative splicing [50]. Our analyses show that the intronic RNA of many genes, including oncogenes, showed significant differential expression after exposure of the cancer cells to antioxidant supplements. RT-PCR validation of expression of introns of DLK1 and LRG1, lung cancer genes verified the results of the bioinformatic analyses. Intronic RNA expression was stronger after NAC supplement treatment in A549 cells, as seen in Figure 4.

Gene Set enrichment analyses of the genes that exhibited changes only in the expression of introns yielded cell cycle, lipid metabolism, and immune system as the top ranking pathways. Similar to our observation, Nakaya *et al.* identified exonic and intronic tissue-specific expression signatures for human liver, prostate and kidney [51]. These investigators reported that the most highly expressed introns were transcribed from introns of protein-coding genes significantly enriched in the “Regulation of transcription” Gene Ontology category. They also showed that RNA polymerase II inhibition resulted in increased expression of a fraction of intronic RNAs in cell cultures, suggesting that other RNA polymerases may be involved in the biosynthesis of intronic RNA.

Gene Set enrichment analyses of genes with differential expression of introns from our analyses on cancer datasets yielded significant enrichment in the immune system. All three antioxidants analyzed in this study, NAC, SFN and Vitamin E influenced immune pathways. Antioxidant supplements are generally considered to boost immune responses and to prevent diseases [52]. However, recent studies show adverse effects of antioxidant supplements in malignancies and tumor progression [53,54]. The novel mechanistic role of antioxidants on the intronic RNA expression of the genes involved in immune system may have a regulatory role in cancer progression.

The expression of introns of several genes that are linked to lung cancer was significantly increased with NAC and Vitamin E treatments (Table 3). Figure 2 shows the schematic representation of RNA-Seq reads from both NAC and Vitamin E treated cells mapping to the intronic region of LRG1. Expression of introns of LRG1 was significantly increased in the antioxidant treated tissues compared to control tumor tissue. LRG1 plays roles in protein-protein interaction, signal transduction, and cell adhesion and development; this gene has been linked to lung cancer [46,47]. According to Liu *et al.*, LRG1 was overexpressed in both the blood level and tumor sections, which can be referred to separate lung cancer patients from healthy cases [55]. Li *et al.* identified LRG1 as a candidate biomarker in urine for non-invasive NSCLC [25]. Increased intronic RNA expression observed for LRG1 in our study may exert a regulatory role on function and expression of the normal LRG1 protein. We were able to validate the expression of introns in two cancer cell types using RT-PCR. Figure 4 shows a strong band for the LRG1 intronic RNA in the vitamin E treated A549 cells, which agrees with our observation in the computational intronic analyses (Figure 2).

Delta-like 1 homolog (DLK1) gene is another example where antioxidant treatment increased intronic RNA expression; the increase was significant with NAC supplement treatment. Furthermore, we validated the intronic RNA expression using RT-PCR. DLK1 gene encodes a transmembrane protein containing six Epidermal Growth Factor repeats; the protein is involved in the differentiation of several cell types. Aberrant expression of DLK1 has been found in various types of human cancers, including lung cancer [24]. Li *et al.* [24] suggest that DLK1 can promote the invasion of lung cancer cells by upregulating MMP9 expression. It is tempting to speculate that the aberrant DLK1 intronic RNA expression may have a role in lung cancer progression.

In this study antioxidant supplements reduced the exonic expression of ROS enzymes including catalase, GPXs and SODs in the murine lung cancer dataset. This was further supported by cell culture experiments showing decreased SOD activity after exposure of cancer cell lines to Vitamin C, Vitamin E and Green tea extract. The observed decrease in endogenous SOD activity could be the result of the antioxidant supplements exerting a negative feedback on the endogenous antioxidant enzyme systems and could negate any potential beneficial effect of the antioxidant supplements.

## 5. Conclusions

In conclusion, this study makes the observation that antioxidant supplements differentially alter the intronic RNA expression of genes including several oncogenes. Furthermore, amongst the cadre of genes comprising the ROS system the change in expression of introns was observed only in SOD gene. The differential intronic RNA expression after supplement treatments, especially the increase observed in oncogenes suggests a possible effect on disease progression. However, further studies are warranted to establish the precise biological significance of this effect of antioxidants on expression of introns.

## Supplementary Materials

The following are available online at www.mdpi.com/2076-3921/5/1/1/s1, Figure S1: Scatter plots showing the number of introns that are differentially expressed with the antioxidant supplement treatments compared to the total number of introns, Supplementary Data 1 and 2: The complete list of genes with differentially expressed intronic RNA.
