# Supplementary material for: Effects of Antioxidants in Human Cancers: Differential Effects on Non-Coding Intronic RNA Expression
Source: Antioxidants (Basel). 2016 Jan 4;5(1):1. doi: 10.3390/antiox5010001 (PMC4808750; doi:10.3390/antiox5010001)
Supplement: Supplementary file 1 [file antioxidants-05-00001-s001.zip › antioxidants-05-00001-supplementary/antioxidants-98973-supplementary figure.docx]

Supplementary Materials: Effects of Antioxidants in Human Cancers: Differential Effects on Non-Coding Intronic RNA Expression

Shreya Menon, Chunxia Lu, Rajasree Menon, Jessica Schwartz and Yuanfang Guan

| 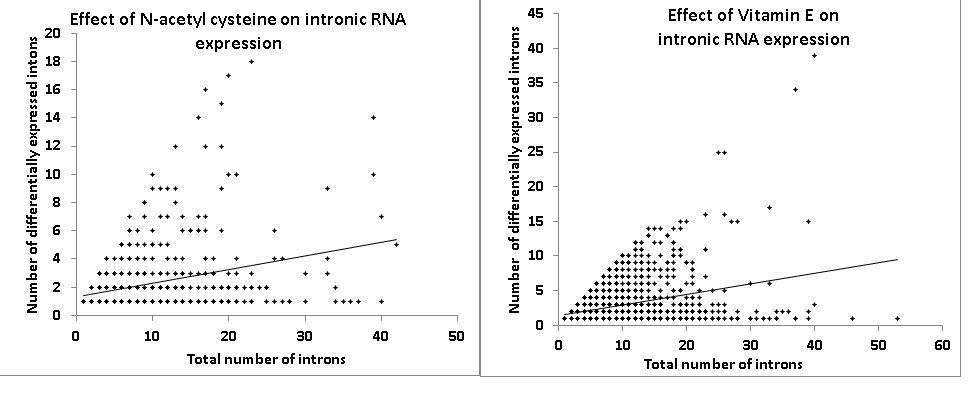 | 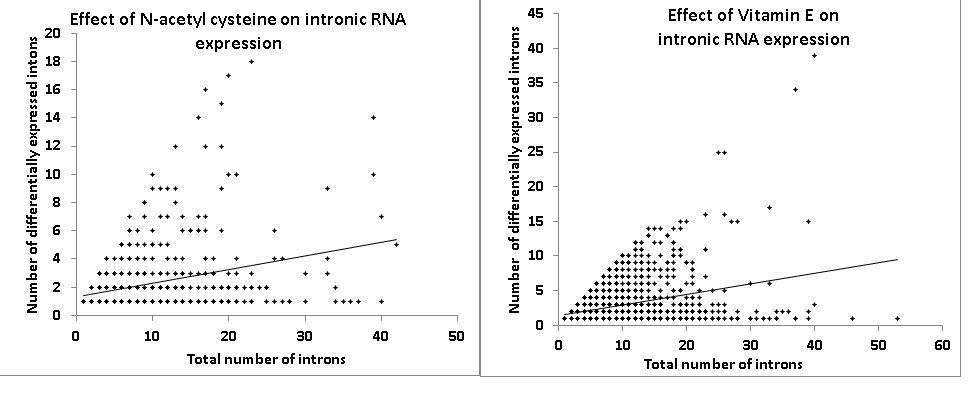 |
| --- | --- |

**Figure S1.** Scatter plots showing the number of introns that are differentially expressed with the antioxidant supplement treatments compared to the total number of introns.
